# The Medical Testimony of Drs. Willard Parker and Gilman in the Trial of Huntington

**Published:** 1857-04

**Authors:** 

**Affiliations:** Brooklyn


### *The Medical Testimony of Drs. Willard Parker and Gilman in the Trial of Huntington.* Analysed by Dr. Braunlich,* of Brooklyn.

Motto: "Non omnia possumus omnes."

The extensive forgeries of Huntington, his trial and subsequent conviction, are of so recent date and so well known, as to render repetition unnecessary. However zealously and energetically the defense might have labored to free the prisoner of his criminal responsibility, no success could have ever crowned their laborious exertions, for the criminal acts of Huntington were too plainly, too evidently and glaringly established by the prosecution, to entertain the slightest hopes for their client.

In fact, nothing was left to them but the plea of insanity, so commonly resorted to under circumstances of so desperate and hopeless a character.

Drs. W. Parker and Gilman were called upon by the defense to inquire into the state of Huntington's mind. Having visited and conversed with the prisoner for about one hour and a half, the medical witnesses did not hesitate for a moment to declare the latter morally insane. Not only did the bench and jury disregard the said medical evidence entirely, but the former rejected it even with signal contempt. Let us inquire whether there was any ground for that action.

The arguments on which the medical evidence chiefly rested are as follows:

1. Huntington manifests a great nervous excitability by scrofulous constitution, in consequence of which the organization of his brain is deteriorated and himself has become insane.

---

* Doctor B. has been one of the most respected and well known physicians for the treatment of Insanity in all its forms. He has been at the head of a large Lunatic Asylum in the Kingdom of Saxony for more than twenty-five years, and his writings on Psychology and Insanity placed him among the most distinguished men of that speciality, Germany possessed. The reason why the Doctor, in advanced age, emigrated to this country, was to obtain the commutation of the sentence of death against one of his beloved sons, who had taken a part in the revolutionary movements at Dresden.

2. Huntington gave away and squanderad the large sums he had obtained by his fraudulent operations in the most senseless manner; proof: that he is insane.

3. Huntington manifested, when in prison, the greatest possible indifference in reference to his own condition, as well as to that of his family; proof: he is insane.

4. In the ancestors, especially in the father and mother of Huntington, some indications of insanity have shown themselves, consequently the insanity is hereditary.

The form of his insanity is that of moral insanity, a species of monomania, by which only the moral functions of the mental faculties are diseased, whilst the others are perfectly sound.

No. 1.—That the scrofulous constitution is apt to produce such disorganizations of the brain as to become the pathological basis of insanity, is as novel as any hitherto unheard-of assertion, a mere hypothesis, borne out neither by science nor experience. Had Drs. Parker and Gilman diligently inquired into the real condition of the insane, they would have found that in these unfortunate patients the scrofulous constitution is by no means prevalent. It is a fact well known to all physicians who have acquired a sufficient degree of knowledge and experience in the treatment of the insane, that no constitution itself predisposes to insanity, or to any particular form of mental disease. Hence the conclusion, that the preponderance of the scrofulous constition of Huntington has directly or indirectly contributed to the insanity of the prisoner, is totally untenable.

No. 2.—Huntington gave away and lavished large sums in the most senseless manner. In this action the medical evidence of Drs. Parker and Gilman will recognise the proofs of his insanity. Had the Drs. been well instructed psychologists, they would have found the direct reverse. The first source of every crime is found in the presence of bad disposition and desires, which men, instead of suppressing, rather favor and nourish. With nutriment of bad dispositions and desires, they grow to passions and degenerate to vices and crimes. This is the ordinary course and developement of every crime of whatever name it may be.

Was this the same case with Huntington? We can give no other than an affirmative answer. His inordinate longing after enjoyment, his tendency to squander, was the first proneness of Huntington that served as the primitive motives to his subsequent criminal acts. His circumstances were not qualified to indulge in them, and in order to satisfy his propensities he resorted to illegal means, thus becoming a criminal. By inquiring into the history of criminals, to which the literature of the "new Pitaval" furnished ample and inexhaustible material, the number of those is rather large that followed the course we have described, and that spent the results of their crimes, from forgery up to murder, in almost the same manner as Huntington.

If Huntington had amassed those sums which he fraudulently obtained, and lavished without indulging in any excess and debauchery whatsoever, the medical evidence would have been much more justified in pronouncing him insane, and consequently irresponsible. As there would have remained no conceivable object for his crime, he might have been laboring under "money-monomania," or with other words, under the insanity of avarice, for which it is well known the mere possession of money is the only object. But even this plea would not be tenable, unless borne out by other unmistakable symptoms of insanity, for avarice is not insanity itself. Huntington, however, was not avaricious, but on the contrary he was a squanderer, and he followed therefore the ordinary course of criminals.

No. 3.—Huntington appeared in prison indifferent and apathic, as to his own future and that of his family. How the medical evidence can find in the apathy and indifference of a prisoner the proofs of insanity is only comprehensible in supposing that Drs. Parker and Gilman have had little or no opportunity at all to observe patients afflicted with insanity. Of hundreds of instances, idiots excepted, we have not found one who had not manifested the greatest uneasiness, restlessness, despair, ire, or violence, or grief and melancholy, when confined to solitude, however kindly and humanely they were treated. The calmness and indifference of Huntington signified therefore the reverse. The prisoner had, in consequence of his enormous expenditures, acquired a certain notoriety in the metropolis; hitherto he had been surrounded by friends and flatterers. His intellectual faculties had been incessantly put on the stretch in one direction; he had been constantly anxious to conceal his forgeries before the world. And at once his whole artificial construction, made up by sham, lie and fraud, broke down; his artificial character was "played out;" no hypocrisy could re-establish him to his former position; the veil fell from his true face, and there stood the defaulter in his criminal nakedness. It is so common in the history of criminals, that a moment like that relaxes the previous excitement into apathy and lethargy, and it is found particularly frequent with criminals belonging to the so-called "higher classes." Herein alone may be found the indifference of Huntington, and hereby again Huntington verified his standing on the common level with criminals.

No. 4.—By Huntington's uncles and aunt there have been traits of insanity; Huntington's mental disease is claimed to be hereditary. It cannot be denied that mental affections descend to the issue through generations; this is, however, much more the case with physical affections and diseases. Supposing that Huntington originated with parents afflicted to a certain extent with mental aberrations, it follows his predisposition to insanity, not insanity itself; just as little as an individual is considered epileptic that has descended from epileptic parents, before the disease has fairly appeared with an epileptic paroxysm; so with Huntington. There is no proof of his being insane, and as yet his alleged hereditory predisposition is merely gratuitous.

The form of Huntington's mental disease Drs. Parker and Gilman define as moral insanity. The nature of this remarkable disease Dr. Parker defines as follows: Moral insanity occurs in an individual who is afflicted with defective organization of some single and certain parts of the brain. The patient may be perfectly self-conscious, and be in the possession of his intellectual faculties; he may be able to discrimate between right and wrong, between good and evil, but in the affected parts of the brain, propensities, inclinations, and impulses may generate, which to balance and to counteract, the patient is not possessed of the required moral power. Justly can we say, "*difficile est satyra non scribere.*" The term of "moral insanity" is in itself a nonentity, a "*contradictio in adjecto,*" and it is almost incomprehensible how a scientific man and well educated physician can use such a term without meaning, and use it under so important and consequential circumstances.

For every act an individual commits in a state of unsound mind, he is irresponsible, because his mental disease deprives him of his moral freedom, and as long as he is in the state of insanity, morality does not exist. If there was moral insanity, there would be necessarily an insane morality, which, however, as yet nobody has thought of. No criminal is at once consummated; he becomes so by degrees, neglecting to suppress by his moral freedom his original faults, propensities, and inclinations; they grow to vice, wickedness, and crime by constantly indulging in and promoting them.

Should the doctrines of Drs. Parker and Gilman gain ground, and be adopted by practical jurisprudence, influencing bench and juries, they would cause a terrible commotion in our criminal justice, and upset all our ideas hitherto entertained by civilized countries, with reference to right and wrong. Every criminal who does not possess the required moral power to restrain his vicious propensities, and had allowed them to develope themselves to vice and crimes, could have a right to set up the plea of moral insanity, and legal guiltlessness therefrom. Dr. W. Parker has hitherto enjoyed the reputation of a highly qualified surgeon, and I do willingly believe deservedly, but sure it is that he is no psychologist, that he is an entire stranger in the science of psychiatrix. And hence he would have done by far better to resign medical evidence in cases refering to the soundness or affections of the mind, to men better acquainted with the subject, and more competent of judging it. It is at the expense of medical dignity and influence, if medical testimony is advanced which bench and juries are obliged to disregard, as they had to do in the case under consideration.—*American Medical Gazette.*

